# Merging Social Networking Environments and Formal Learning Environments to Support and Facilitate Interprofessional Instruction

**DOI:** 10.3885/meo.2009.T0000132

**Published:** 2009-04-28

**Authors:** Sharla King, Elaine Greidanus, Michael Carbonaro, Jane Drummond, Steven Patterson

**Affiliations:** University of Alberta, Edmonton, Alberta, CANADA

**Keywords:** Collaboration, Online communication, Interprofessional education, e-learning

## Abstract

This study describes the redesign of an interprofessional team development course for health science students. A theoretical model is hypothesized as a framework for the redesign process, consisting of two themes: 1) the increasing trend among post-secondary students to participate in social networking (e.g., Facebook, Second Life) and 2) the need for healthcare educators to provide interprofessional training that results in effective communities of practice and better patient care. The redesign focused on increasing the relevance of the course through the integration of custom-designed technology to facilitate social networking during their interprofessional education. Results suggest that students in an educationally structured social networking environment can be guided to join learning communities quickly and access course materials. More research and implementation work is required to effectively develop interprofessional health sciences communities in a combined face-to-face and on-line social networking context.

## Introduction

Reforms to health service delivery currently focus on teamwork and collaboration. The Canadian Health Services Research Foundation Policy Report for Teamwork in Healthcare highlighted that “a healthcare system that supports effective teamwork can improve the quality of patient care, enhance patient safety and reduce workload issues that cause burnout among healthcare professionals” (pp. iii, Executive Summary).^1^ The framework document entitled *Interprofessional Education for Collaborative Patient-Centered Practice* highlighted the necessity of educating health professionals in collaborative practice skills alongside clinical skills early in their education.^2^

The increased focus on teamwork and collaboration among health professionals does not necessarily mean team members are spatially or temporally present in practice. The use of innovative technologies to facilitate health teams’ working together is an increasing trend.^3^ However, teams using technology to communicate must become fluent in the use of that technology to sustain and expand the benefits of effective interprofessional teamwork. This requires that graduating Health Science students must possess a combination of disciplinary clinical skills, interprofessional team skills, and a fluency in the use of information technology to aid collaboration for improved patient care.

Health science students’ fluency with information technology may already put them at a distinct advantage over many practicing health professionals. Social networking in virtual spaces is currently the norm for many university students, with 90% of undergraduates actively involved in social networking.^4^ Rather than disregard these online social networking skills students already possess, universities should assist students to harness the professional collaborative opportunities provided by the on-line tools. To ensure that interprofessional teams can collaborate effectively in future clinical settings, healthcare students must learn how to communicate in both face-to-face and virtual environments. Given these challenges, universities must integrate technology into educational environments to address learner needs, prepare students to work and function in blended (virtual and face-to-face) communities of practice upon graduation,^5^,^6^ and investigate the development and effectiveness of these educational environments.

At the University of Alberta, the Health Sciences Council was created in response to the evolving healthcare environment.^7^ For the past 12 years an interprofessional course has provided Health Science students with an opportunity to participate in interprofessional student teams within which they develop collaboration skills. Previous research in this course investigated the development of collaborative skills in a face-to-face small-group learning environment.^8^ This paper describes the redesign of this team development course to increase its clinical relevance and to support the use of virtual learning communities. It also reports the initial findings about students’ behaviors in the context of this redesigned learning environment.

**Communities of Practice** - Communities of practice create the capacity to share and acquire knowledge to solve complex problems in dynamic and evolving team problem-solving environments. The term “communities of practice” was first used in the early 1990s by Lave and Wenger^9^ to describe a mode of learning based on an apprenticeship model. A community of practice is a group of people who share an interest in a specific domain and then engage in collective learning that creates a bond among them.^10^ Therefore, acquiring knowledge in order to solve complex problems is a social process in which individuals learn through practice. The key to cultivating a community of practice is the development of a collaborative learning environment in which individuals share and create knowledge. In the healthcare field, communities of practice are increasingly utilized for continuing professional education, knowledge management and information sharing.^6^,^10^,^11^ The internet's dominant use is now the sharing of information and communication rather than functioning as a vast reference source, thus creating virtual communities. As a result, virtual communities of practice are emerging as possible mechanisms to address the increasing challenges of health service delivery and to provide a means for health professionals to manage their work in complex and often fragmented contexts.^3^,^12^,^13^

**Figure 1.**
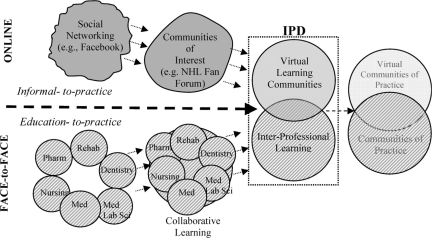
Model of the merging of informal technology skills with formal professional skills.

To nurture the development of communities of practice in a digital world, students need the opportunity to engage in instructional experiences that seamlessly operate in both face-to-face and online environments. Such integrated educational environments are often referred to as *blended learning*.^14^ Figure 1 represents a model demonstrating the merging of informal technology skills with formal professional skills, creating a space in which students develop professional, technology-based communication skills.

The top portion of Figure 1 represents the progression of students’ social networking skills. As indicated by the top left circle, most students first engage in informal technology-based social networking, such as Facebook^15^–^17^ instant messaging, and email with friends and family. These informal networks are associated with informal social networking skills such as online abbreviations (LOL for “laughing out loud,” BRB for “be right back,” and sideways faces [:) or ?] known as emoticons to indicate the emotional tone of a comment). Individuals with these skills and common interests combine to form communities of interest such as a discussion group about a computer game or popular music group. As technology-based tools are integrated into education systems, students can begin to use their informal social networking skills to create virtual learning communities such as those described in this paper. As these students move into professional practice, they can continue to use these skills in formal ways to facilitate communities of practice in health care. Therefore, the top portion of Figure 1 indicates a progression from *informal-to-practice* social networking.

The bottom portion of the diagram represents the progression of face-to-face *education-to-practice.* Health sciences students traditionally receive their education in discipline specific “silos” (Medicine, Nursing, Pharmacy, Medical Laboratory Science, Dentistry, and Rehabilitation Medicine). Educational institutions may formalize collaborative and interprofessional learning experiences with the expectation that students develop effective team skills, better preparing them for clinical practice. As students enter into the workforce, they form networks or connections with other professionals, across the disciplines, thus promoting the emergence of interprofessional communities of practice.

The top and bottom portions of Figure 1 are partially separated by a dashed arrow indicating the developing intersection between online and face to face (F2F) experiences as they apply to developing online communities of practice. The ability to work and function in virtual learning communities may better prepare students to work and function in virtual communities of practice. The *Interprofessional Desktop* (IPD) section of the diagram represents a learning community designed to take advantage of students’ existing online social networking experiences to develop online collaboration for professional practice purposes. Figure 1 shows how the virtual learning community plays an important role in the seamless integration of online and F2F activities during an interprofessional educational experience. This model was used as a framework for redesigning the interprofessional course and introducing technology and collaborative tools.

**The Interprofessional Team Development Course** - The goal of the course is to develop appropriate team process skills in an interprofessional environment. Students learn about the different health disciplines, their interrelationships, and establishing ongoing collaborations with other professions. The course emphasizes working in a team to complete a task. Twelve years ago the course began as an option with approximately 25 students from a variety of disciplines. Currently, it is a required course for 9 Health Science programs with approximately 800 students enrolled annually (Nutrition, Medicine, Dentistry, Dental Hygiene, Nursing, Pharmacy, Physical Therapy, Occupational Therapy and Medical Laboratory Science). It is optional for students in the Faculty of Physical Education and Recreation.

Each interprofessional team includes at least one student from Nursing, Medicine and Pharmacy, as those programs have large student cohorts. Students from other programs are distributed across the teams to ensure that no more than one member of each discipline is on each team. Each team has an average of 6–8 students. Within each classroom, 6 teams are overseen by at least 2 facilitators (a faculty member and a clinical practitioner). The facilitators’ role is to guide the students through the activities by providing feedback and assistance. The class is offered during 5 weeks. It is a full credit course (32.5 hours); students therefore attend classes 3 hours on Tuesdays and 3 1/2 hours on Thursdays. Both afternoon and evening sessions are offered due to the large number of students involved.

The teams work through case scenarios with the emphasis on recognizing unique and similar contributions of the different professions while maintaining a patient/client-centered approach. There are 5 key team-process concepts covered in the course: personal and team reflection, giving and receiving feedback, consensus decision making, conflict resolution, and team roles. Each scenario emphasizes one concept, but all concepts are integrated to various degrees throughout the course. A typical case scenario is a video-based presentation of a complex geriatric client admitted to the hospital after a fall. The teams role-play an admission and discharge conference with standardized patients playing the client and family. The scenario's objective is that students discuss discipline-specific information and integrate that information into an interdisciplinary care plan for the client. A more complete overview of the course is provided by Taylor et al. (2004).^8^ Studies of the course over the past several years indicate that students from all disciplines enhance their team-process skills.^18^–^21^

## Course redesign in the context of the University of Alberta

Student feedback about the interprofessional team course indicated that students would prefer the learning content and course format maximize clinical relevance. Based on this feedback, the University of Alberta's Health Science Faculties identified two areas on which to focus the course redesign. First, information and communication technologies were integrated to enhance instruction and to facilitate the development of professional virtual communities of practice. These technologies included course management software (WebCT), virtual classroom technologies (Elluminate), and a link to our social networking IPD environment. Second, instructors emphasized self-directed learning by organizing the course around areas of clinical interest associated with a clinical site visit.

**Collaborative Technology** - Technology features were integrated to facilitate course management, information management, and learning community technologies in the form of the custom designed IPD system (see Figure 1). IPD was developed by University of Alberta's Centre for Health Evidence (http://www.cche.net/default.asp) in collaboration with the Health Sciences Council. The IPD was modeled on a technology platform similar to existing systems being used by healthcare professionals.

**Figure 2.**
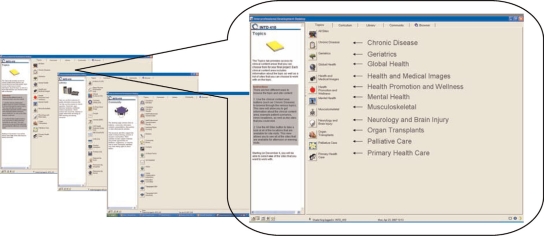
Examples of images from Interprofessional Desktop (IPD).

The student orientation to the IPD and the course format occurred 6 weeks prior to the course's start. Students received a brief introduction to the redesigned course and instructions for accessing the desktop. Two primary areas in the IPD environment were the Curriculum and Community windows. Figure 2 shows the screen interfaces for IPD. The Curriculum window presented students with the class schedule, agenda (objectives for each class), and team process information (e.g., processes for resolving conflict, giving/receiving feedback or reflection). The Community window contained the collaborative tools such as message boards and the team's shared files and was the area where students could quickly connect and share information with their teammates and facilitators.

**Clinical Teams based on Common Interest** - An important feature of the course redesign included students’ selecting into a team based on areas of common clinical interest rather than random selection. Therefore, prior to the start of the course, students used the IPD to select an area of clinical interest (see Figure 2). These clinical interest areas were used to form learning communities with common foci and to determine which clinical site the team would visit. Students’ self-selection of interest areas was a significant redesign enhancement to the course and directly based on the model outlined in Figure 1. These pre-structured interest contrast with the freely subscribed interest areas of social networking environments such as Facebook that naturally develop around users. Allowing for an open-ended or pure discovery-learning approach is often not well suited to novice learners,^22^,^23^ particularly in technological environments.^24^ Furthermore, our model is based on merging open-end online social networking with formal collaborative learning environments. For students to develop collaborative team-based problem solving skills in a technologically driven social networking environment, structural pedagogical supports are important.

## Results

Over 620 (91% of the course's students) students signed into the IPD and selected a content area during the first week they were eligible, 30 days prior to the start of class. This suggests that students were motivated to identify their areas of interest early and felt comfortable enough with the IPD technology to use it to select their clinical area of interest. The self-selected, small-group content areas that were filled first were Global Health, Musculoskeletal, Organ Transplants and Chronic Disease. Table 1 shows the breakdown of students by disciplines.

**Table 1.** Distribution of students by disciplines

Disciplines	#Student
Dental Hygiene	40
Dentistry	32
Medical Laboratory Science	24
Medicine	130
Nursing	98
Nutrition	51
Occupational Therapy	84
Pharmacy	129
Physical Education and Recreation	25
Physical Therapy	72

**Figure 3.**
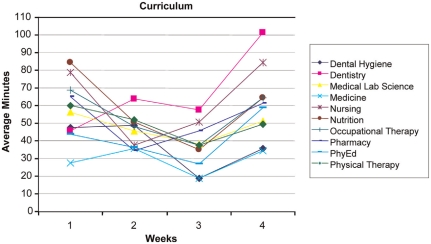
Graph of IPD usage of the curriculum resources (average minutes/student accessed) over the first 4 weeks of the course.

**Figure 4.**
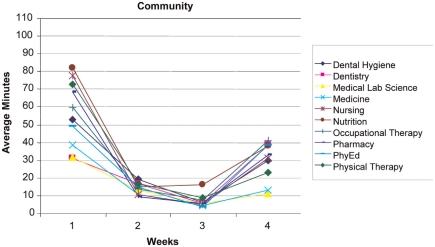
Graph of IPD usage of the community resources (average minutes/student accessed) over the first 4 weeks of the course.

**Use of the IPD** - Examination of usage reports revealed that the number of hours the students used the IPD technology each week dropped substantially after the first week and each subsequent week (Figures 3 and 4). During Week 1, students accessed the desktop for 225 hours collectively. In weeks *2-A* usage dropped to between 78–86 collective hours. At Week 5 the usage dropped further to 43 hours. Week 5 was not included in the figures because team presentations were occurring in the class. The IPD was not utilized as fully as the course re-designers had anticipated. However, patterns of IPD use can be examined to help assess the course redesign and to indicate areas for further improvement. This result indicates that students were initially motivated to access the IPD but were not motivated to continue using the IPD throughout the 5 weeks. This result may also indicate that students’ previous experiences with social networking predisposed them to use the IPD but, finding the IPD cumbersome (i.e., lacking an easy-to-use Facebook-type interface), they stopped using it when they did not need to.

During the first week of class, the most frequently accessed part of the desktop was the course Curriculum area (549 hits, 41.4 hours). The Community area was the next most frequently accessed area (301 hits, 16.1 hours). Figures 3 and 4 show the time usage trend across all 9 disciplines. With respect to the Community tool (Figure 4), usage was higher in the first week, dropped for weeks 2 and 3, and then began to rise on week 4. This use pattern supports the course instructors’ observation that student team members were initially motivated to use the IPD to connect with other students and access resources, but there was less need to use collaboration resources when students met face-to-face twice a week. The rise in use toward the end of the course may indicate an increase in use related to greater familiarity. This slight increase toward the end of the course may support the theory that an online community takes time to develop and that the community may have further developed if the course had continued past 5 weeks.

**Discipline-Specific Trends** - Overall, the averages for student access to the desktop by disciplines ranged between 3 hours a week for Medicine up to 7 hours a week for Nutrition. Examination of the usage data distribution revealed that the frequent desktop users (n = 25) accessed the desktop from 29.7 to 51.5 hours over the 5 week period. A subset of Pharmacy students (n = 7) had the highest usage numbers. Subsets of Nursing and Occupational Therapy students (each n = 5) were next. Despite having one of the larger student cohorts, only a single medical student was included in the frequent users group. This may indicate that Medical students find IPD less relevant than those from the other professions.

**Figure 5.**
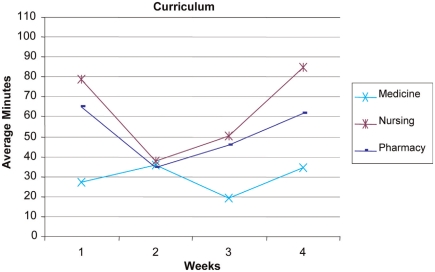
Graph of IPD usage of the curriculum resources (average minutes/student accessed) for students from Medicine, Nursing and Pharmacy over the first 4 weeks of the course.

**Figure 6.**
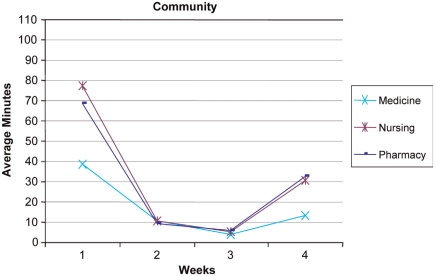
Graph of IPD usage of the community resources (average minutes/student accessed) for students from Medicine, Nursing and Pharmacy over the first 4 weeks of the course.

We continued our analysis in the IPD areas of Community and Curriculum focusing on Medicine, Nursing and Pharmacy (see Figures 5 and 6) for 3 reasons. First, the other 6 disciplines followed the Nursing and Pharmacy trends across both areas. Second, a frequent collaboration exists among these 3 disciplines as demonstrated by the Alberta Provincial Colleges of Medicine, Nursing, and Pharmacy joint conference entitled Strengthening the Bond: Collaborating for Optimal Patient Care, (2007). Finally, the number of students in each of the 3 groups was relatively close. A 3-way ANOVA was conducted to assess the differences in curriculum time over the 4-week period. Significant differences were found in weeks 1, 3 and 4 between Medicine and both Nursing and Pharmacy. Significant differences were noted for week 1 [F = 7.63, df = 2,502; p B .05], for week 3 [F = 4.19, df = 2,502; pB.05], and week 4 [F = 6.18, df = 2,502; p B.05]. These results indicate that students from Medicine, Nursing, and Pharmacy had distinctly different patterns of IPD use throughout the course. Overall, Medicine students demonstrated a consistently lower usage pattern for IPD resources as compared to students from other professions. The Nursing and Pharmacy students accessing the Curriculum section showed a similar use pattern that was initially high, dropped, and then increased again at the end of the course.

**Lessons Learned in Preparation for Future Redesigns** - The collaboration between the IPD developers and course coordinators illustrates the complexities of collaboration. The main focus was heavily weighted to the initial course launch and student self-selection into small groups. Not enough discussion was put into some of the processes for the course. For example, curriculum management relied on templates and processes that were optimal for complex and long-term educational interventions rather than a 5-week course. Student communication processes using the desktop were not established clearly at the onset. Therefore, once the course was underway the logistical challenges prevented development of a process.

The logistical complexities of incorporating technology into a 5-week course for 800 students also provided a number of challenges during the course preparation phase. Facilitators were unable to become fully fluent with the IPD themselves before the course began because the IPD was not fully completed by the time the facilitator training began. Formal IPD training was also not provided to the students in the course. Although the course redesign team had considered providing more formal training, they decided against it because the IPD designers believed that the IPD design was intuitive and would not require formal training. Instead, the students were given an information sheet that directed them to an online tutorial. However, the students were not required to complete the tutorial; they generally did not find the IPD intuitive or were unwilling to negotiate the various IPD components. Some student comments reported that the IPD had “too many layers,” was cumbersome, and had icons that were too small. Some students had difficulty accessing the IPD from their home computers and in some computer labs on campus. Many students reported that they stopped using the IPD because it became “too frustrating.” This finding indicates that the transition from informal to professional use of technology needs to be carefully streamlined in order to facilitate students’ professional learning.

Based on student and instructor feedback, in combination with the IPD use results, the course designers identified a number of areas for future improvement. First, the course instructors will participate in more thorough IPD training prior to the course's beginning. This early training will focus on familiarity with the IPD technology as well as establishing a culture of online learning. This will include facilitating the process of primarily communicating with students via the IPD instead of face-to-face. Students will also be provided with a training session to familiarize them with the IPD, its purpose and navigation of the various tools and components. Course templates and tools will also be reworked, decreasing pressure on course instructors to adapt the material and facilitating the instructors’ abilities to focus on seamlessly integrating the course design to the IPD. Future courses will formally emphasize the relevance of interprofessional health care technologies such as IPD in the healthcare system rather than having the focus of the technology appear to be curriculum-management driven. For example, including more’ dummy’ electronic health records will increase the IPD's relevance for students and may enhance motivation to use the IPD. The IPD itself will be redesigned to be a more intuitive format similar to widely used social networking systems such as Facebook or MySpace. Additionally, a major course-schedule change will extend the course to 10 weeks to provide more time to develop the professional online community.

## Conclusions

This paper described how one interprofessional team development course at the University of Alberta began to address the complex collaboration and communication challenges facing future health professionals. The model suggested in this paper describes how informal technology-based social networking can facilitate a transition to professional, collaborative health care. The model is based on evidence that indicates social networking is a pervasive phenomenon among today's university students and that such networks can result in the creation of social capital.^4^,^25^ The model assumes that social networking, combined with face-to-face classroom training, provides a basis for developing effective interprofessional communities of practice leading to better patient care. This assumption requires continued investigation about how students use and integrate new informal types of social technologies with professional training and collaboration. Further research is also needed to understand how technology-mediated communication for interprofessional teams should be structured to facilitate growth of effective communities of practice and to investigate the usage patterns of discipline specific cohorts.

Initial results highlight the importance of continually adapting technologies to provide students with the skills required to practice in an ever changing healthcare system. The custom-created IPD system that was implemented as part of the research work is one such technology. Online communication skills are slowly emerging as a key competency for health professionals to assist in overcoming distance and time challenges in providing care. The development of these skills should commence during pre-licensure education so graduates enter clinical practice with a solid foundation for collaborating in face-to-face and virtual practice environments. The overall goal is to develop educational models that will result in improved clinical practice and patient care. Finally, this paper emphasizes the importance of clinical relevance in student learning. Specifically, students need to clearly understand why and how technologies are relevant to clinical practice if educators want students to maximize their use within a course.
